# Non-Panel Practitioners

**Published:** 1913-08-30

**Authors:** 


### THE NATIONAL MEDICAL GUILD.

President: CHAS. BUTTAR, M.A., M.D., B.C.

Gen. Sec.: P. C. RAIMENT, M.R.C.S., L.R.C.P.

Offices: 34 Villiers Street, Strand, London, W.C.

### NON-PANEL PRACTITIONERS.

We have spoken of the need for the unity of the profession and for the setting up of one common platform on which we could again all meet and present a united front in the case of any future attacks. The introduction of the National Insurance Act has unfortunately divided the profession into two broad classes, those of the panel and those not of the panel. This is the division that is apparent to the general public, but as a matter of fact in these two divisions there are many grades, and if a remedy is to be found for our unfortunate differences a proper understanding of these sub-divisions is above all things necessary.

### The Non-Panel Practitioners' Outlook.

Those not of the panel will not detain us long. They fall at once into three classes: (*a*) the consultant who, if not unaffected, is not the person who will ever do the class of work demanded by the Government; (*b*) the general practitioner in a high-class general practice who again is unlikely to take on any Insurance work; and (*c*) the general practitioner who would, if the terms and conditions were what he considered fair and reasonable, accept this class of work, and by his refusal to do so is probably sustaining loss every day. The first two classes may be dismissed from our minds at once. Neither of them is likely to be any the worse off for the introduction of the Act, and they are quite content to pursue their profession without any help from anybody. But when we come to the third class, of whom there are hundreds, we are concerned with a grave problem. These men whose sense of honour is so acute that they cannot accept the release of the pledge that they willingly gave, command at once our admiration and our sympathy. They are a tower of strength to the profession, and will be of the greatest assistance to us in any future trouble, because we know that their word once given will never be departed from. At the same time it is a matter of regret that they should be daily penalised for their loyalty to the profession. If the penalty were to stop at the loss that they have already sustained the harm done would not be so great, but we fear that the loss must be an ever-increasing one. We commend the following argument to them. Assuming that any such are established in practice in a quasi-industrial area, their record of services in the past will stand them in good stead for the present, and we will assume that the families of the insured will not desert them for any importation, even if the insured worker transfers his allegiance to the Insurance doctor. But the same will not hold good for all time. In any industrial area in London the population is of necessity a floating one, and the newcomers will not have the same allegiance to the non-panel doctor, and will probably drift quite easily into the hands of the panel doctor if his rank and standing in the neighbourhood be fairly good, and so for every old patient that leaves the district they cannot reckon on replacing with a newcomer, as they have lost the introduction that the panel gives them, and so year by year the industrial side of their practices must dwindle, until in the fullness of time they become a negligible quantity, and the doctor be compelled to exist on the fees of his good patients. If these are present in sufficient numbers for him to make a living out of, well and good. But if they are not the result must be the annihilation of the doctor. From this argument one might be tempted to think that we advocate such men as these going to the panel. Not at all. It will be our endeavour to give them such help as will enable them to keep off, and unless some help is given, and moreover given speedily, these men must go to the wall or join the panel. We will return to the remedy next week.

### THE SCHEMES OF (a) THE MEDICAL GUILD AND (b) THE BRITISH MEDICAL ASSOCIATION.

### The Special Strength of a Medical Trade Union.

**What Dr. Fothergill Wishes to Know.**

*To the Editor of* The Hospital.

Sir,—In your issue for the 23rd inst., page 626, you are good enough to find space for certain questions I desired to submit to the President of the National Medical Guild. Immediately following are certain paragraphs put forward as the answers of that Guild. Now, in those questions there is no mention whatever of the British Medical Association, and yet the "answers" are in most cases almost entirely confined to discussing the actions of that body.

No doubt your numerous readers, therefore, will be disappointed. They will have hoped from the answers for some enlightenment; some hint, to say the least, as to the power behind a *medical* trade union that so far had escaped their knowledge. They did not wish to hear about the Association. Are they to conclude that the National Medical Guild acknowledges that after all there is no greater power in a *medical* trade union than in a *medical* association—no special power to prevent blacklegs obtaining or retaining appointments, or employers (private or public) from obtaining medical attendants; no special power to prevent individuals or groups bargaining; no special power to maintain a standard rate or for making certain of the payments of levies or fines? In fact, do they own that the power, so far as there is any, will depend in the last resource on the honourable conduct of the medical members to the Guild and one to the other?

If there will be a special power in a *medical* trade union not possessed by the British Medical Association, is it asking too much of the President of the National Medical Guild to disclose it to your readers by answering each of the questions submitted to him by your courtesy? I am certain he would not wish us medical practitioners to join in ignorance.—I am, etc.,

E. Rowland Fothergill.

Brighton, August 23, 1913.

What will the B.M.A. Scheme Cost?

P.S.—Then another and entirely separate subject to which I must return seeing I have been challenged to do so. In your issue of August 16, on page 398, the National Medical Guild criticised a scheme of the British Medical Association and stated that it would cost 13s. 4d. to collect every £1. I said that this was a misstatement of fact. That this is so is obvious if the scheme is carefully read. It is proposed to use the fund of £50,000 in providing clerks, solicitors, medical organisers, offices at Edinburgh, Dublin, and Cardiff, payment for attendance at committees, etc., etc. This will use the 13s. 4d. in each £1 collected, the balance going to a reserve fund. *But these expenses will not be incurred unless and until the money is forthcoming.* The actual clerical and other expenses incurred in trying to collect the £50,000 should be easily covered by £1,000. Therefore, to say that "it will cost 13s. 4d. to collect £1" is undoubtedly a misstatement of fact. Let us hear, however, now about the medical trade union and its special and peculiar powers. E. R. F.

### IMPORTANT ISSUES FOR MEDICAL PRACTITIONERS.

### THE GUILD'S REPLY:—A PHANTOM FUND.

We regret that Dr. Rowland Fothergill in his letter of this week has relegated to the obscurity of a postscript the question of the cost of administering the proposed fund of the British Medical Association. We were under the impression that he charged us with making a misstatement of fact. This is a grave allegation and the subject merits more publicity than Dr. Fothergill seems to want to give it. Can the explanation be that Dr. Fothergill desires to drop the matter, and, if so, why? Again we must press home the fact that this phantom fund is designed to keep a phantom army of clerks, secretaries, treasurers, and what not, and that for every sovereign that the profession subscribes 13s. 4d. is devoted to expenses. We have had occasion to make this remark before, but we make no apology for repeating it. It is the truth, unpalatable as Dr. Fothergill finds it, and the importance of this truth we shall continue to press upon all and sundry in season and out of season.

### Inducements to join a Medical Trade Union.

All the questions that Dr. Fothergill has put to us can be summed up in one sentence, viz., How can you induce medical men to join a trade union, and, having joined, how can you keep them as members? The "special powers" that Dr. Fothergill refers to we will deal with later. The answer to his question is not easy. It is one that has defied solution by every body ever founded to help members in regularising wages, terms of contract, etc. The British Medical Association has failed to answer the question, and it still remains to be seen whether the National Medical Guild will be any more successful. To seek the answer to the first part of the question one must go back to primary causes. What induces a man to do anything? Either the desire to benefit himself or the desire to amuse himself, or to gratify one of the senses. No one would join a trade organisation for the latter reason, but if you can show him that he will derive material benefit either now or in the future he may come in. For example, many join the British Medical Association solely to obtain the *Journal* week by week. If then the Guild can give its members some material advantage they will join, and so long as that advantage is kept up they will remain in.

### How the Union may retain its Members.

The answer to the second half of the question is that the large majority of men are willing to remain loyal to any association so long as its policy is in accordance with their interests. Such men will require no coercion. The problem then remains how to deal with the minority, who will always break away the moment they see any personal advantage in so doing. At the present moment there is no body but a trade union that can deal effectively with this portion of the profession, and if these men can be made members they can be held. For instance, no man desires to be expelled publicly from his professional organisation especially if that body has gained any repute. Further, if he is so expelled (and note that he cannot resign at a period during which a dispute is in progress), and desires to rejoin a heavy penalty is enforced. Removal from the union is a heavy weapon if wielded properly, but in order to make it effective it must be to the member's advantage to remain inside, otherwise removal loses its terrors. Briefly then the plan of the Guild is, Make it to the advantage of the medical man to be a member and all else follows. We have discussed in earlier numbers how we propose to do this, and to them we must refer Dr. Fothergill.

### Special Powers Vested in Trade Unions.

Now a word as to the special powers possessed by trade unions. The power of unionism depends upon the immunity that trade-union officials enjoy in committing acts, denied to ordinary people, in the furtherance of their propaganda. Also, more important still, the absolute protection and immunity granted to their funds. As Dr. Fothergill seems rather doubtful of this first point we will quote him the section of the Act in question: "An act done by a person in contemplation or furtherance of a trade dispute shall not be actionable on the grounds only that it induces some other person to break a contract of employment or that it is an interference with the trade, business, or employment of some other person, or with the right of some other person to dispose of his capital or his labour as he wills." Let us interpret this in a concrete instance. In the future let us assume that some fresh and unpalatable enactment is suddenly laid upon the profession by this or some future Government. What remedy has an ordinary organisation? It can say that after January 15 next the profession will refuse to renew its contract unless the defect is remedied. If the profession were all in a trade union it would only be necessary to inform the Government that unless the offending clauses were eliminated at once the profession would refuse to go on with its contracts and no action would lie. The power to determine a contract without notice is a very valuable weapon.

Now, with regard to the funds. If there were no other reason this should be sufficient to drive the profession to accept trade unionism at once. A union's funds are protected by the Friendly Societies Act, 1855, and the Trade Union Act, 1869, and that protection is absolute. Not so the funds raised by an association. If anyone disbelieves this let him read the Supplement to the *Journal* of July 5, wherein he will find the opinion of the legal advisers of the British Medical Association. To conclude with the point with which we commenced we may say that, having regard to that opinion, we feel glad that most of the proposed fund of the British Medical Association is to be spent on organisation as the balance is certainly liable to attachment.

### THE MEDICAL GUILD AND THE B.M.A.

As we go to press we have received an interesting communication on this subject, which we regret to have to hold over until our next issue.

